# Composition of the Schistosoma mansoni worm secretome: Identification of immune modulatory Cyclophilin A

**DOI:** 10.1371/journal.pntd.0006012

**Published:** 2017-10-26

**Authors:** Achilleas Floudas, Christopher D. Cluxton, Julia Fahel, Adnan R. Khan, Sean P. Saunders, Sylvie Amu, Antonio Alcami, Padraic G. Fallon

**Affiliations:** 1 Trinity Biomedical Sciences Institute, School of Medicine, Trinity College Dublin, Dublin 2, Ireland; 2 Centro de Biología Molecular Severo Ochoa, Universidad Autónoma de Madrid, Madrid, Spain; University of Edinburgh, UNITED KINGDOM

## Abstract

The helminth *Schistosoma mansoni* modulates the infected host’s immune system to facilitate its own survival, by producing excretory/secretory molecules that interact with a variety of the host’s cell types including those of the immune system. Herein, we characterise the *S*. *mansoni* adult male worm secretome and identify 111 proteins, including 7 vaccine candidates and several molecules with potential immunomodulatory activity. Amongst the molecules present in the secretome, a 17-19kDa protein analogous to human cyclophilin A was identified. Given the ability of cyclophilin A to modulate the immune system by regulating antigen presenting cell activity, we sought to determine whether recombinant *S*. *mansoni* Cyclophilin A (rSmCypA) is capable of modulating bone-marrow derived dendritic cell (BMDC) and T cell responses under *in vitro* conditions. rSmCypA was enzymatically active and able to alter the pro-inflammatory cytokine profile of LPS-activated dendritic cells. rSmCypA also modulated DC function in the induction of CD4^+^ T cell proliferation with a preferential expansion of Treg cells. This work demonstrates the unique protein composition of the *S*. *mansoni* male worm secretome and immunomodulatory activity of *S*. *mansoni* Cyclophilin A.

## Author summary

Helminths are known for their ability to alter the host’s immune response in order to promote their survival. One such mechanism is the propensity of helminths to secrete molecules with immunomodulatory activity; such molecules alter various aspects of host immunity to the benefit of the parasite. Following detailed characterisation of the secretome, we have identified that Cyclophilin A is secreted from adult *Schistosoma mansoni* and that the protein has the capacity to alter dendritic cell and T cell function *in vitro* by inducing a T cell regulatory phenotype.

## Introduction

Schistosomiasis is one of the most prevalent parasitic diseases, with approximately 230 million people being infected globally [1]. To develop improved schistosomiasis control strategies, including new drugs and vaccines, it is important to advance our understanding of how the parasite manipulates the host’s immune system to achieve the chronic infection state that is characteristic of helminth infections in man. The trematode *Schistosoma mansoni* is the second most common schistosome species responsible for cases of schistosomiasis in the tropics and subtropics [2, 3].

The co-evolution of parasitic helminths and mammals has led to the development of a spectrum of mechanisms whereby the immune system of the infected host is bypassed or modulated to facilitate the completion of the parasite’s life cycle [4, 5]. The mechanisms that govern the host’s immune system modulation during helminth infection include the release of excretory/secretory (ES) products from the helminth, that for an example drive a ‘modified’ T cell response as a mechanism of suppression of the immune system, to regulate protective host responses and in addition, prevent immunopathology [6, 7]. *S*. *mansoni* infection evokes a spectrum of cytokines, such as IL-4, IL-5, IL-10, IL-13, IL-25, IL-33 and TGF-β, as well as modulating the function of various immune cells, including regulatory T (Treg) and B cells, eosinophils, alternatively activated macrophages and tolerogenic dendritic cells (DC) [8, 9]. Indeed, helminth ES contains potent immunomodulatory molecules (IM) that have activities beyond their function in helminth immunity, and have been explored as potential therapeutic molecules [10–13].

Helminth infection modify the functions of T cells with the generation of Th2 CD4^+^ T cell responses and expansion of Treg cells to evoke a state of helminth-induced T cell hypo-responsiveness [9, 14–17]. These effects are not only caused by the direct activity of ES molecules on T cells, but also indirectly, through modulation of antigen presenting cell (APC) activity [18]. DCs form a heterogenic network of cells comprised of several subsets that are capable of responding to a variety of danger signals [19]. Interactions with certain pathogen pattern recognition receptors, including toll-like receptors (TLRs), drive DC maturation and antigen presentation, with elevated MHCII and co-stimulatory molecules (CD80, CD86) expressed on their surface. Additionally, TLR ligands can differentially influence DC cytokine production with LPS stimulation enabling pro-inflammatory cytokine (IFN-γ, IL-6 and IL-12) secretion [20]. In the course of a helminth infection, and despite the availability of TLR ligands, which include helminth-secreted components, DC subtypes demonstrate altered activity, with impaired cytokine release, decreased expression of co-stimulatory molecules and altered antigen presentation capacity that induces the development of a hypo-responsive T cell response [21].

*S*. *mansoni* male worm infections of mice induce a state of modified immunity *in vivo*, rendering mice refractory to anaphylaxis, allergic lung inflammation and experimental colitis [22–25]. Therefore, we sought to characterise the worm excretory/secretory (WES) proteome of adult male *S*. *mansoni* worms, and analyse the immunomodulatory potential of WES to identify novel anti-inflammatory IM and vaccine candidates. In the secretome, seven known vaccine candidates were present, and several potential IM were detected, including a secreted non-classical cyclophilin A (SmCypA). Enzymatic activity of the generated recombinant SmCypA (rSmCypA) was assessed, and the effect of SmCypA on bone marrow (BM) derived DC was investigated with regards to BMDC activation in response to TLR stimulation. We also determined the effect of rSmCypA on DC antigen presentation and subsequent T cell proliferation *in vitro*. Collectively, we demonstrate that the *S*. *mansoni* secretome has a unique composition that includes potential vaccine candidates and IM as well as a human CypA analogue. SmCypA modulates DC leading to the preferential expansion of Treg cells *in vitro* that may contribute to T cell hypo-responsiveness during *S*. *mansoni* infection.

## Methods

### Mice and infections

C57BL/6J and OT-II (TCR^OVA^) transgenic mice were from Jackson Laboratory (Maine, USA) and bred in-house. All mice were bred in a specific pathogen-free barrier facility with male mice used at 8–10 weeks of age. A Puerto Rican strain of *S*. *mansoni* was maintained by passage in mice and albino *Biomphalaria glabrata* snails, as previously described [26].

### Ethics statement

All animal care and experimental procedures were performed under an Irish Department of Health and Children Licence (holder Padraic Fallon, Licence Number B100/3250) in compliance with Irish Medicine Board regulations. Animal experiments received ethical approval from the Trinity College Dublin Bioresources Ethical Review Board (Reference: 121108).

### Preparation of male adult worm excretory-secretory (WES) and adult worm (AW) molecules

Mice were infected with 200–300 *S*. *mansoni* cercariae and portally perfused, to recover worms, six to seven weeks after infection, as described previously [26]. Mice were perfused in Minimum Essential Media (MEM) supplemented with Earle’s Salts (Gibco) and 26.2 mM sodium bicarbonate and further washed several times; male worms were then selected via microscopic examination and any damaged, stunted or dead male worms were discarded. Male worms were subsequently washed thoroughly with 10 mL RPMI-1640 supplemented with 2 mM L-glutamine (Gibco), 50 IU Penicillin/Streptomycin (Gibco) and 5 μg/mL Gentamicin (Gibco) at 37°C under sterile conditions. Two hundred male worms were transferred to cellulose membrane dialysis tubing with a 10 kDa molecular weight cut-off (MWCO; Thermo Scientific) in incubation media; RPMI-1640 supplemented with 2 mM L-glutamine, 100 IU Penicillin/Streptomycin and 5 μg/mL Gentamicin. The tubing was placed in a T-75 cell culture flask containing nutrient media, incubation media with 10% Foetal Bovine Serum (FBS; Sigma) for up to 72 hrs (**S1 Fig**).

Worms were incubated at 37°C for seventy-two hours; the nutrient media was changed after 24 and 48 hours of incubation. Worm incubation media was harvested and concentrated using a stirred cell concentrator at 50 psi with a 10 kDa MWCO membrane (Amicon). The concentrated supernatant was dialyzed with Dulbecco’s Phosphate Buffered Saline (PBS; Biosera) at 4°C using a 10 kDa MWCO dialysis cassette. The WES preparation was then centrifuged and filtered through a 0.22 μm filter. All WES batches were subjected to a quality control analysis (QCA). A batch is defined as a specimen containing the concentrated WES products from 200 male worms over 72 hours, pooled together and stored at -80°C.

AW molecules were prepared as described previously [27]. In brief, live male worms were isolated, as above, and disrupted under liquid nitrogen with a percussion mortar. The resulting paste was sonicated and centrifuged (10,000 × g) for 1 h at 4°C. The supernatant was repeatedly clarified using a micro-centrifuge at 4°C followed by filtration through 0.45 μm and 0.22 μm filters. AW was stored at a stock concentration of 1 mg/ml at -80°C.

QCA entailed a sample from a batch being resolved on a 12% sodium dodecyl sulfate polyacrylamide gel (SDS-PAGE) and visualized by silver staining. Serum from WES immunised rabbit was used in comparative Western blot analysis. Protein and endotoxin levels were assessed by BCA (Thermo Scientific) and LAL assays (Thermo Scientific), respectively.

### WES protein separation, detection and mass spectrometry

300 μg of WES or AW proteins were precipitated using 10% w/v trichloroacetic acid (TCA; C_2_HCl_3_O_2_) and resuspended in rehydration buffer (8M urea, 40 mM Tris, 4% CHAPS, 2% v/v IPG Buffer, 0.002% w/v Bromophenol blue, 60 mM dithiothreitol; DTT). For first dimension separation, WES/AW products were loaded onto 13 cm IPG strips, pH 3–11 (GE Life Sciences), followed by reduction with 1% w/v DTT and alkylation with iodoacetamide (IAM, 2.5% w/v). Second dimension separation was performed on a 138 (W) x 130 (H) mm 12% SDS-PAGE using the ATTO Corporation electrophoresis system AE-6220 (ATTO Bioscience and Biotechnology). Running buffer was added to the buffer chamber and the gel was run at 25 mA and stained by Coomassie Brilliant Blue (Thermo Scientific). All visible spots were manually collected and identified by mass spectrometry.

Mass spectrometry was performed by the BSRC Mass Spectrometry and Proteomics facility at St. Andrews University (http://www.st-andrews.ac.uk/~bmsmspf/). The samples were analysed by a quadruple-time-of-flight mass spectrometer, the Q-STAR Pulsar XL (Applied Biosystems). Briefly, samples were digested with trypsin and loaded onto a capillary liquid chromatography system (nanoLC system). The peptides were separated by reverse phase chromatography and directly eluted into the mass spectrometer. The peptides were then subjected to electrospray ionization and tandem mass spectrometry (ESI-MS/MS) generating a mass (m) -to-charge (z) ratios (m/z) spectrum. The mass spectrometry data was analysed using the Mascot software (http://www.matrixscience.com/) searching the *S*. *mansoni* GeneDB sequence database (http://www.genedb.org/Homepage) for protein hits. Protein hits were identified through peptide mass fingerprints. Protein hits that showed at least 2 matched peptides with expectation values lower than 0.05 were considered positive hits.

### Bioinformatic analyses of WES

The analysis for protein homologues was performed using the Protein-Protein Basic Local Alignment Search Tool (BLASTp) accessed at the National Centre for Biotechnology Information (NCBI) website (http://blast.ncbi.nlm.nih.gov/Blast.cgi). Sequences alignments that showed a bit score higher than 30 and an expected value (*E*-value) lower than 1*e*^-16^ were considered homologues. Analysis of Gene Ontology (GO) was performed using the QuickGo web-based browser provided by the European Bioinformatics Institute (EBI, http://www.ebi.ac.uk/). GO terms associated with biological process and molecular function were addressed to each protein sequence by searching the UniProtKB-GOA database. Protein sequences were subjected to analysis by the web-based browser SignalP 4.0 Server (http://www.cbs.dtu.dk/services/SignalP/) for predictions of a secretory signal peptide. Protein sequences that did not contain a signal peptide were subjected to analysis by the web-based browser SecretomeP 2.0 Server for prediction of non-classical secretion *i*.*e*. not signal peptide triggered protein secretion. SecretomeP analysis was performed using predictions for mammalian sequences and proteins that showed a SecP score higher than 0.5 where considered non-classically secreted proteins. The TMHMM Server v. 2.0 (http://www.cbs.dtu.dk/services/TMHMM/) was used for the predictions of transmembrane helices in the protein sequences. The InterProScan (http://www.ebi.ac.uk/Tools/pfa/iprscan/) sequence search was used for assignment of protein signatures. This tool combines different protein signature recognition methods that search against specific databases. The protein signatures that describe the same protein family or domain are grouped into unique InterPro entries, with a unique accession number. Alignment of multiple sequences was performed using the ClustalW2 programme provided by the EBI (http://www.ebi.ac.uk/Tools/msa/clustalw2/).

### Recombinant *Sm*CypA production, purification and validation

For the production of rSmCypA a construct was designed to incorporate an N-terminal Honeybee melittin signal peptide and a C-terminal polyhistidine (His) affinity tag. Recombinant protein was expressed *via* baculovirus infection of SF9 insect cells and purified by nickel-NTA and size exclusion chromatography. Endotoxin was removed by Triton-X washing, with rSmCypA having <0.01 EU/mg as assessed by LAL assay.

For confirmation of purity, 5 μg of protein was loaded onto SDS-PAGE gels followed by western transfer for detection of the His-tag. 15% SDS-PAGE gels were visualized using Coomassie Brilliant Blue. For western transfer, proteins were immobilized onto a PVDF membrane (Millipore) at 30V for 18 hours at 4°C. Protein transfer was confirmed via Ponceau S (Sigma) staining. Membranes were blocked and probed with HisProbe-HRP (1:5000 dilution; Thermo Scientific) in Odessey Blocking Solution (Li-cor) for 1 hour. Membranes were developed using ECL Western Blotting Substrate (Thermo Scientific Pierce) and visualized via the ChemiDoc MP system (Bio-Rad). Gel images were acquired via HP Scanjet G4050.

The peptidyl-prolyl *cis*-*trans* isomerase (PPIase) activity of rSmCypA was measured using protease-coupled spectrophotometric assay [28], which follows the cleavage of the *trans* form of the chromogenic peptide substrate, *N*-succinyl-Ala-Ala-Pro-Phe-4-nitroanilide (Suc-AAPF-pNA; Sigma) by chymotrypsin. The serine protease inhibitor PMSF was added to reactions to block proteolytic activity. Reactions were carried out at 23°C in 200 μl of assay buffer (35 mM HEPES buffer, pH 7.9, 86 mM NaCl, and 0.015% Triton-X-100). Ice-cold chymotrypsin solution (added from a 2 mM stock prepared in 10 mM HCl; Sigma) was added immediately followed by rSmCypA and 4mM of substrate to initiate the reaction. The *cis-trans* isomerization of the Pro-Phe bond was measured by following the absorbance increases at 405 nm over 0–600s using a microplate reader (VersaMax tunable microplate reader; Molecular Devices, Sunnyvale, CA).

### Detection of *SmCypa* expression by PCR

Following lysis of 200 adult male or female *S*. *mansoni* worms using Trizol reagent (Invitrogen), total RNA isolation was performed using the RNeasy kit (Qiagen) and was followed by reverse transcription with the Quantitect reverse transcription kit incorporating a genomic DNA elimination step (Qiagen), as per the manufacturer’s instructions. PCR for the detection of *SmCypa* and housekeeping gene (*Pai1*, *Gapdh*) expression was performed using the following primers, *SmCypa* (forward primer: ACGTCTATGCCACTGACGAC, reverse primer: ATGTGTCAGGGTGGCGATTT), *Pai1* (forward primer: TAGCTCCGACAGAAGCACCT, reverse ACGACCTCG ACCAAACATTC), *Gapdh* (forward primer: ATCCCAGCCTTCGCATCAAA, reverse primer: CATCCCGTGGGATAAGGACG). Fold expression change was calculated by performing densitometry on images of agarose gel electrophoresis for *SmCypa* and housekeeping genes PCR product bands using ImageJ v1.51.

### Bone marrow derived dendritic cell (BMDC) generation and culture

Femur bones were isolated from male C57BL/6 mice and BM was then flushed out using a 27G needle with RPM1-1640 (Gibco). Erythrocytes were lysed using PharmLyse solution according to manufacturer’s instructions (BD Biosciences), and the remaining cells were washed and counted. Cells were subsequently seeded at 2 x 10^6^ cells/ml in untreated petri dishes in complete media (CM; RPMI-1640 supplemented with 10% heat-inactivated Fetal Bovine Serum (Sigma), 2 *m*mol L-glutamine, 50 IU/ml penicillin and 50 μg/ml streptomycin) supplemented with 20 ng/ml GM-CSF (R&D Systems). BM cells were cultured as described in detail by Lutz *et al*., [29]. Following 9 days of culture, 85% of the non-adherent cells expressed the DC marker CD11c (Clone #HL3; BD Biosciences) as assessed by flow cytometric analysis. For functional assays, 1 x 10^6^ BMDCs per ml were treated or not with various concentrations of rSmCypA followed by stimulation for 24 hours with 100 ng/ml ultra-pure LPS (Invivogen) with or without rSmCypA.

### CD4^+^ T cell culture

Spleen and lymph nodes (LN) were harvested from TCR^OVA^ mice and processed to generate single cell suspensions as described previously [30]. CD4^+^ T cells were isolated by magnetic bead negative selection (CD4^+^ T cell Isolation Kit II; Miltenyi Biotec) via AutoMACS. Cell purity was determined to be > 90% via flow cytometry. Cells were then labelled with Cell proliferation dye (eBioscience) as per manufacturer’s instructions before being utilized downstream. For T cell culture, 1 x 10^5^ TCR^OVA^ CD4^+^ T cells were cultured with 2 x 10^4^ BMDC previously treated or not with LPS and/or rSmCypA in the presence of 10μM OVA.

### OVA_323-339_ uptake

To determine OVA_323-339_ uptake in BMDCs, OVA peptide (Cambridge Research Biochemicals) was labeled with the AlexaFluor647 microscale protein labeling kit (Life Technologies) as per manufacturer’s instructions. Isolated BMDCs, as described above, were treated with labeled peptide at 1μM with or without the presence of 100 ng/ml LPS. Cells were analyzed via flow cytometric analysis after 24 hours of incubation with labeled OVA for AlexaFluor647 positive cells.

### Cytokine analysis

Concentrations of IL-12p70, TNF-α, IL-10, IL-4, IL-17A, IFN-γ (R&D Systems) from cell culture supernatants were determined via ELISA as per manufacturer’s instructions. Following Streptavidin-HRP treatment, ELISAs were developed using TMB substrate solution (eBioscience). Absorbance at wavelength 450 nm was read using a microplate reader (VersaMax tunable microplate reader; Molecular Devices).

### Flow cytometric analysis

Single-cell suspensions from in vitro cultures or harvested LNs were analysed by flow cytometry. Cells were washed in flow cytometry staining buffer (PBS with 2% FCS and 0.02% sodium azide) followed by blocking with anti-mouse CD16/32 (2.4G2; BD Bioscience). The following mAbs from BD Biosciences, CD4-PE/V450 (RM4-5), CD80-PE-CF594 (16-10A1), CD40-PE (3/23), eBioscience; CD3-PE-eFluor-610 (145-2C11), CD11b-PerCP-Cy5.5/PE-Cy7 (M1/70), CD11c-PE/PE-Cy7 (N418), MHC-II-FITC/eFluor-450 (M5/114.15.2), Biolegend; PD-L1-PE/APC (10F.9G2), DEC-205-APC (NLDC-145), and Miltenyi Biotec; CD86-FITC/PE (PO3.3) were used at optimally titrated concentrations. For transcription factor staining, cells were fixed and permeabilized with a commercial transcription factor staining kit (eBioscience) according to the manufacturer's instructions, and the cells were stained with the following transcription factors from BD Bioscience—FoxP3-PE/ PE-CF594 (MF23), T-bet-FITC/APC (O4-46), RORγt-BV421 (Q31-378) and eBioscience GATA3-PE (TWAJ). Viable cells were distinguished using LIVE DEAD Aqua (Life Technologies). Populations of interest were gated according to appropriate “fluorescence minus one” controls. Samples were acquired on a CyAn ADP flow cytometer (Beckman Coulter) and were analyzed with FlowJo software (Tree Star).

### Statistical analysis

Data are expressed as mean ± SEM and were analyzed by two-way analysis of variance (ANOVA) test or unpaired Student's *t*-tests (Prism 6; GraphPad Software). Significance for all statistical tests was shown in figures as *P* < 0.05 (*), *P* < 0.01 (**), *P* < 0.001 (***) and *P* < 0.0001 (****).

## Results

### Characterization of the S. mansoni worm secretome

A method for the collection of WES from live adult male *S*. *mansoni* worms *in vitro* [26] was adopted for bulk collection of WES (**S1A Fig**). Only adult male worms were utilized in WES preparations, to exclude any IM secretions from eggs that would contaminate the WES if female worms were included. Several independent batches of concentrated WES were analysed using one-dimensional SDS-PAGE to determine the spectrum of proteins being produced. The protein content of the WES batch was measured, with protein degradation and overall protein profile assessed. There was consistency, with little identifiable variation between the independent batches of WES (**S1B Fig**). WES Batches that showed no protein degradation, a similar protein profile of positive bands using rabbit polyclonal anti-WES antibody (**S1C Fig**) and a maximum of 0.5 endotoxin units per mg (EU/mg) were approved for further analysis. Protein yields were estimated to be equivalent to 110 ng of protein per worm within a 72-hour incubation period. Despite attempts to increase yield through elongated culture times there was little effect on the final protein yield.

**Fig 1:**
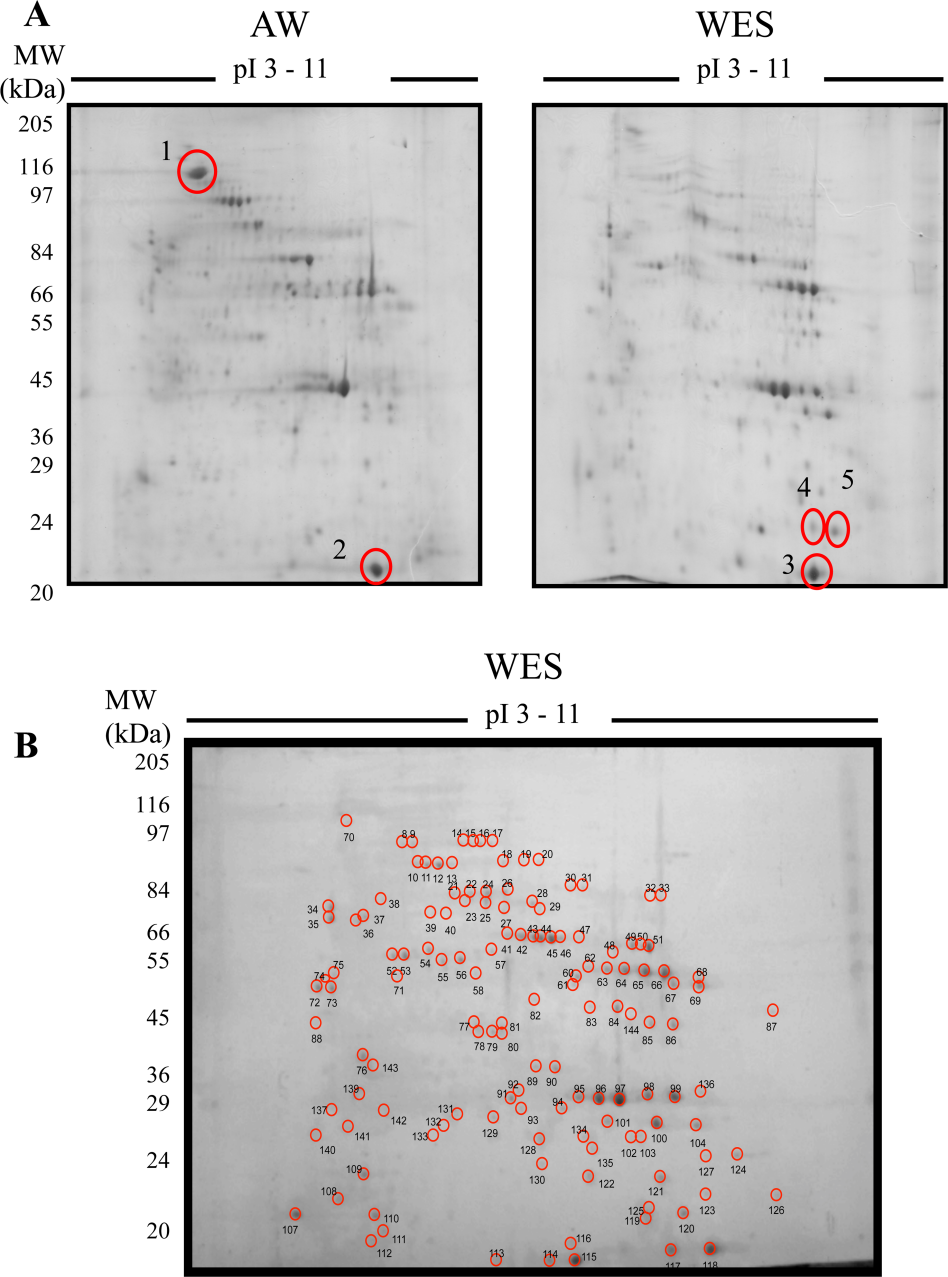
Proteomic analysis of WES molecules. (**A**) Comparative 2D proteomic analysis between adult male worm somatic molecules (AW) and WES molecules. AW and WES (200 μg) were electrofused in a 3–11 IPG strip and then electrophoresed in a 12% SDS-PAGE stained with colloidal-coomassie blue. Five spots (circled and numbered 1–5) were selected for identification by mass spectrometry. Protein hits: 1 –SmParamyosin (Smp_021920.1); 2–3 –SmFatty acid-binding protein (Smp_095360.1); 4–5 –SmCyclophilin (Smp_040130). (**B**) A representative gel of *S*. *mansoni* WES molecules, analysed as in (**A**), showing individual spots that were analyzed by mass spectrometry.

To address the question of whether male adult *S*. *mansoni* worms selectively secrete a unique proteome, we compared WES to a soluble adult male worm homogenate (AW) by two-dimensional (2D) SDS-PAGE. AW and WES preparations were resolved in parallel. The overall distribution and intensity of the spots revealed a differing protein composition between the WES and somatic preparations (**Fig 1A)**. The AW preparation appears markedly more complex, supporting the hypothesis that the parasite actively secretes/excretes a specific fraction of molecules. To confirm this visual demarcation, one spot unique to the AW preparation (spot 1), two spots common to both preparations, with similar isoelectric points (pI) and molecular weights (spots 2 and 3), as well as two spots unique to the WES preparation (spots 4 and 5) were selected for identification by mass spectrometry (**Fig 1B**). Spot 1, which is unique to AW preparation, was identified as *S*. *mansoni* Paramyosin (Smp_021920.1), a major structural protein of schistosomes and other invertebrates. Spots 2 and 3, which are common to WES and AW preparations, were identified as *S*. *mansoni* Fatty acid-binding protein (Smp_095360.1). *S*. *mansoni* fatty acid protein, also known as Sm14, was previously identified in the worm tegument and the *S*. *japonicum* homologue had previously been detected in the adult worm excretory-secretory proteome [31]. Spots 4 and 5, which are unique to the WES preparation, were identified as *S*. *mansoni* Cyclophilin (Smp_040130), also known as Smp17.7. These results demonstrate that WES and AW preparations have common and unique molecules therefore demonstrating a clear demarcation of the protein profile between these preparations.

To further identify the proteins present in WES, selected spots were picked from 2D gels and subjected to electrospray ionization and tandem mass spectrometry (ESI-MS/MS). A total of 170 spots were subjected to analysis by mass spectrometry, of which 136 (80%) showed positive hits when analysed against the *S*. *mansoni* GeneDB sequence database, resulting in the identification of 111 proteins (**S1 Table**). Homologues of some of the *S*. *mansoni* WES proteins identified were also present in two other human-infecting schistosome species, *S*. *japonicum* and *S*. *haematobium* as determined by BLAST analysis. *S*. *japonicum* homologues were detected for all *S*. *mansoni* WES proteins, with more than 70% of the homologues (80 proteins) detected having a similarity to their respective *S*. *mansoni* homologues higher than 80% (**S2 Table**). Furthermore, several schistosome antigens have been previously tested as vaccine candidates in different animal models [32]. A number of vaccine candidates were identified in our analysis of the *S*. *mansoni* WES proteins (**Table 1**). Three out of six potential candidates selected by the WHO in a study in the 1990’s were identified; TPI [33], Sm28GST [34] and Sm14 [35]. The ECL (200 kDa protein) [36], Sm21.7 [37], Sm-p80 [38] and Cu-Zn superoxide dismutase [39] were also detected. Two promising candidates, more recently tested, Sm29 [40] and Sm-TSP-2 [41] were not identified among WES molecules. Sm29 [40] and Sm-TSP-2 [41] have been characterized as membrane proteins highly expressed in the schistosome tegument and therefore are not expected to be excretory-secretory proteins [40].

**Table 1:** Vaccine candidates detected in the *S*. *mansoni* adult male worm secretome.

ID	Protein description	Vaccine name	Vaccine type	Reference
Smp_003990	Triosephosphate isomerase, putative	TPI	Transfer of anti-TPI monoclonal antibody	[33]
Smp_017730	200-kDa GPI-anchored surface glycoprotein	ECL (200 kDa protein)	DNA	[36]
Smp_054160	Glutathione S-transferase 28 kDa (GST 28) (GST class-mu), putative	Sm28GST	DNA	[34]
Smp_086480	Antigen Sm21.7, putative	Sm21.7	Recombinant protein	[37]
Smp_095360.1	Fatty acid binding protein	Sm14	Recombinant protein	[35]
Smp_157500	Calpain (C02 family)	Sm-p80	DNA vaccine + recombinant protein boost	[38]
Smp_176200.2	Cu-Zn superoxide dismutase	Cu-Zn superoxide dismutase	DNA	[39]

ID–identification number at GeneDB.

WES molecules were analysed for predicted signal peptides, both classical N-terminal and non-classical internal. A small number of proteins had a classical signal peptide (6.3%; 8/111) while the majority of signal peptide containing proteins were non-classical (41.4%; 46/111). These proteins were classified as secretory. Three WES molecules were predicted to be trans-membrane (2.7%; 3/111) and the remaining contained no signal peptide (47.7%; 53/111), and were classified as excretory. The ratio however, of secreted to non-secreted proteins may have been influenced by such predictive software optimised using mammalian, rather than parasitic proteins [42].

*S*. *mansoni* WES proteins were screened for the presence of immunomodulatory candidates through homology analysis with other helminth proteins with known immunomodulatory activity (**Table 2**). Based on this analysis, five *S*. *mansoni* WES proteins were considered as potential immunomodulatory candidates: Calreticulin auto-antigen homologue precursor, Serpin, Peroxiredoxin 1 and two Cyclophilin proteins. *S*. *mansoni* Calreticulin auto-antigen homologue precursor contains a signal peptide and Serpin and Peroxiredoxin are predicted to be non-classically secreted with a SecP score of 0.601 and 0.597, respectively. The *S*. *mansoni* Cyclophilin A protein is predicted to be secreted via a non-classical pathway, while Cyclophilin B contains a signal peptide. Based on strong sequence homology we selected SmCypA to examine further for immunomodulatory activity.

**Table 2:** *S*. *mansoni* adult male WES proteins with immune-modulatory homologs.

*S*. *mansoni* WES		Immunomodulatory helminth homologs				
ID	Protein description	GenBank ID	Description	Species	Immunomo—dulatory activity	Ref.
Smp_030370: Calreticulin autoantigen homolog precursor, putative		AAR99585.1	Calreticulin-like protein	*Haemonchus contortus*	Binds to complement C1q inhibiting the classical complement pathway	[43]
		CAL30086.1	Calreticulin precursor	*Heligmosomoides polygyrus*	Th2-skewing property	[44]
		CAA07254.1	Calreticulin	*Necator americanus*	Binds to complement C1q inhibiting the classical complement pathway	[45]
Smp_090080	Serpin, putative	AAB65744.1	Serpin precursor (Bm-spn-2)	*Brugia malayi*	Specific inhibition of neutrophil proteinases cathepsin G and neutrophil elastase	[46]
Smp_059480	PeroxiredoxinPrx1	AAB71727.1	Peroxiredoxin	*Fasciola hepatica*	Induction of alternatively activated macrophages	[47]
Smp_040130	Cyclophilin A	EPT31956.1	Peptidyl-prolyl cis-trans isomerase	*Toxoplasma gondii*	Interacts with CCR5 receptor in DCs and Mϕ	[48]
Smp_040790	Cyclophilin B, putative					

ID–identification number at GeneDB or GenBank databases.

### Production of recombinant SmCypA

**Fig 2:**
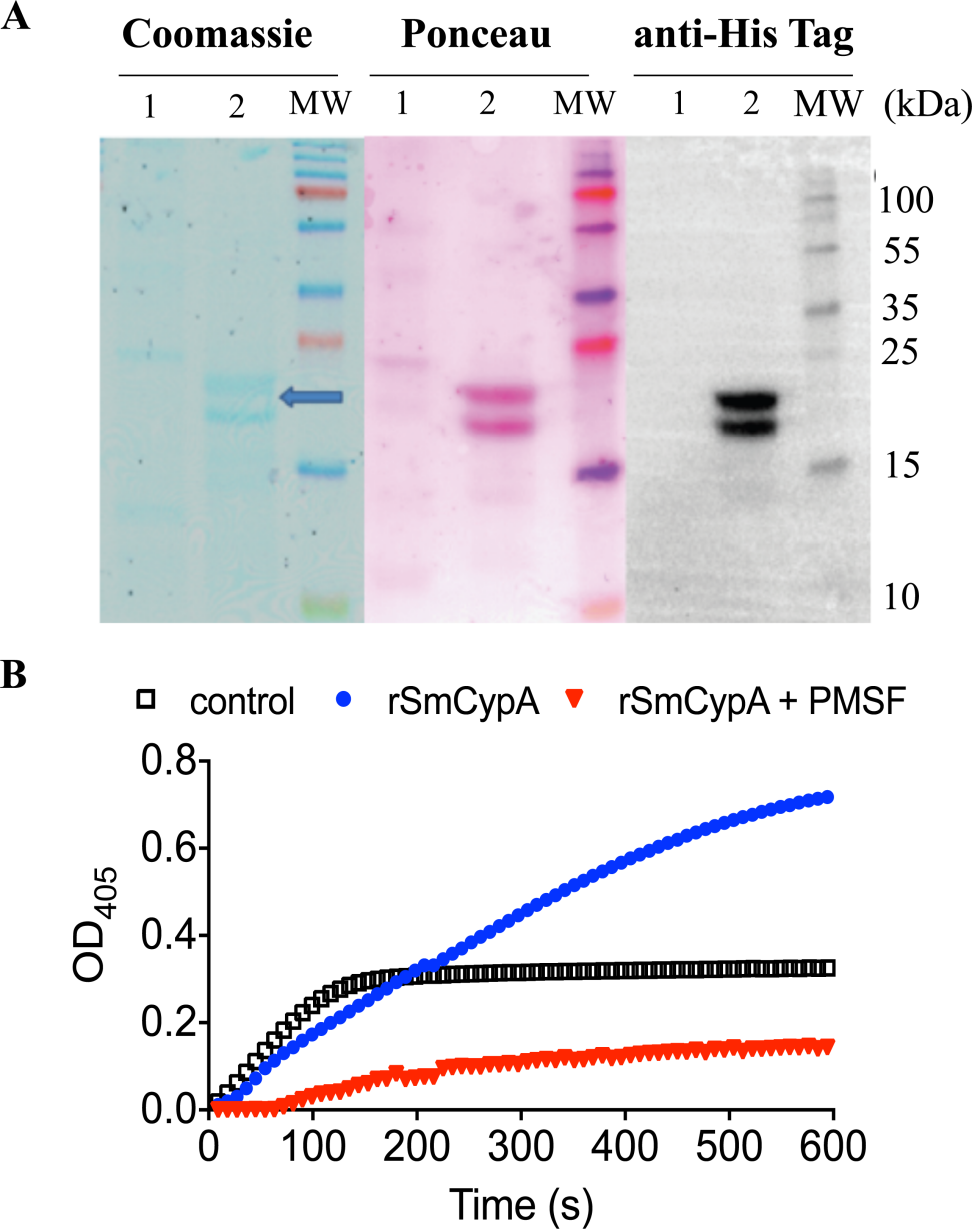
Production of rSmCypA. **(A)** Coomassie stain of SDS-PAGE gel containing WES **(Lane 1)** and rSmCypA **(Lane 2),** with a subsequent Ponceau stain following western transfer and Anti-His tag expression to identify recombinant protein. Molecular Weight (MW) markers are shown. (**B**) Quantification of PPIase activity of rSmCypA in the presence or absence of PMSF. Figure is representative of three independent experiments.

rSmCypA was expressed in insect cells and after Nickel and size chromatography purification, two bands, with a molecular weight of 18–22 kDa, which corresponds to predicted characteristics of the cyclophilin family [49, 50] were detected, following coomassie staining of SDS-PAGE resolved proteins. Western blot, using an anti-His tag, confirmed the predicted size of the recombinant protein (**Fig 2A**). To determine if rSmCypA was enzymatically active, the *cis* to *trans* isomerization of succinyl-Ala-Ala-Pro-Phe-4-nitroanilide was measured using the standard protease-coupled assay. rSmCypA was found to have PPIase activity, 30 μg/min, with increased Suc-AAPF-pNA cleavage to levels above that of the control reaction of chymotrypsin-α alone (**Fig 2B**). Furthermore, by inhibition of the signal using the chymotrypsin-α inhibitor phenylmethanesulfonylfluoride (PMSF), the PPIase activity was determined to be chymotrypsin-α dependent (**Fig 2B**). Therefore, the recombinant SmCypA protein generated purified as an enzymatically active protein.

### SmCypA alters BMDC function and LPS activation

**Fig 3:**
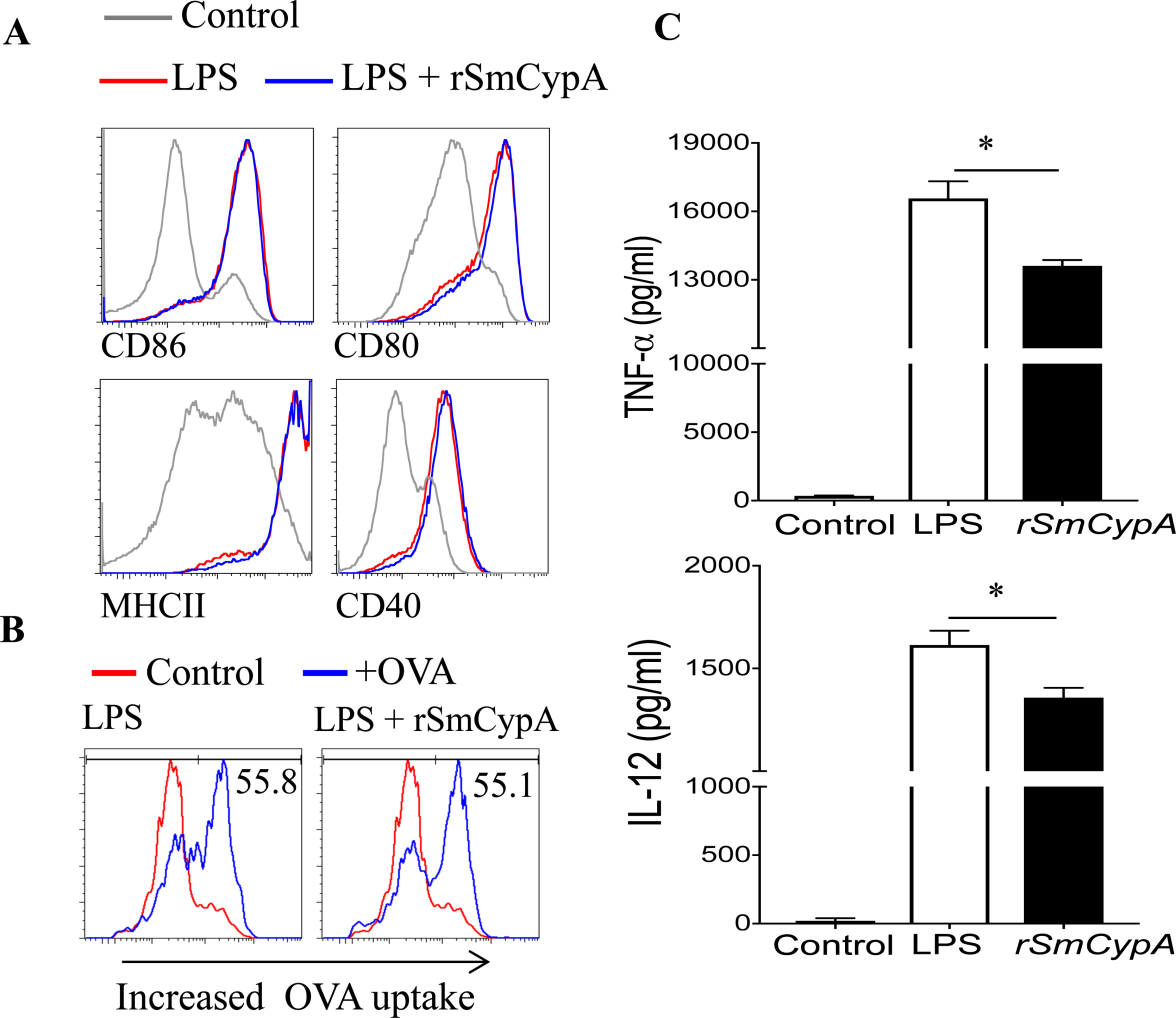
Immune modulatory effects of rSmCypA on BMDC. (**A**) Representative histograms from flow cytometric analysis of LPS treated and LPS + rSmCypA co-treated BMDC for cell surface expression of CD86, CD80, MHCII and CD40. Data shown are representative of 5 independent experiments. (**B**) Representative histograms of BMDC for Alexa Fluor-647 labeled OVA_323-339_ uptake following LPS induced stimulation with or without simultaneous treatment with rSmCypA. Data shown are representative of 2 independent experiments. (**C**) ELISA for the quantification of TNF-α and IL-12 in the supernatant of BMDC treated with LPS only or co-treated with LPS and rSmCypA, n = 3 per group. Data shown are representative of 2 independent experiments. Data are presented as mean and SEM and statistical difference between groups was determined using Student's *t* test.

Given that cyclophilins from other species have been reported to modulate BMDC function [49], we sought to demonstrate whether rSmCypA has a similar activity. TLRs have been shown to play a key role in the recognition of helminth products by the host, and certain helminth products have been shown to modulate the activation of DC in response to LPS activation [51, 52]. Therefore, BMDC were treated with rSmCypA during simultaneous activation with LPS for 24 hours. Representative example of gating strategy for flow cytometry analysis of BMDC and the effect of LPS treatment on DC activation is shown (**S2 Fig**). Treatment with rSmCypA during LPS induced activation did not have an effect on cell surface expression levels of several molecules (CD80, CD86, MHCII, CD40) involved in DC antigen presentation (**Fig 3A**).

We then assessed the capacity of rSmCypA treated BMDC to uptake antigen. For that purpose, OVA_323-339_ was labeled with Alexa Fluor-647 and antigen uptake by LPS-only treated or LPS activated and rSmCypA co-treated BMDC was assessed by flow cytometric analysis. The uptake, by DC, of antigen and presentation capacity of labeled OVA_323-339_ was not altered following co-treatment with LPS and rSmCypA (**Fig 3B**). Interestingly, despite the inability of rSmCypA to alter BMDC cell surface expression of several co-stimulatory molecules, rSmCypA altered BMDC pro-inflammatory cytokine production, resulting in significantly (P<0.05) reduced expression of TNF-a and IL-12 (**Fig 3C**).

**Fig 4:**
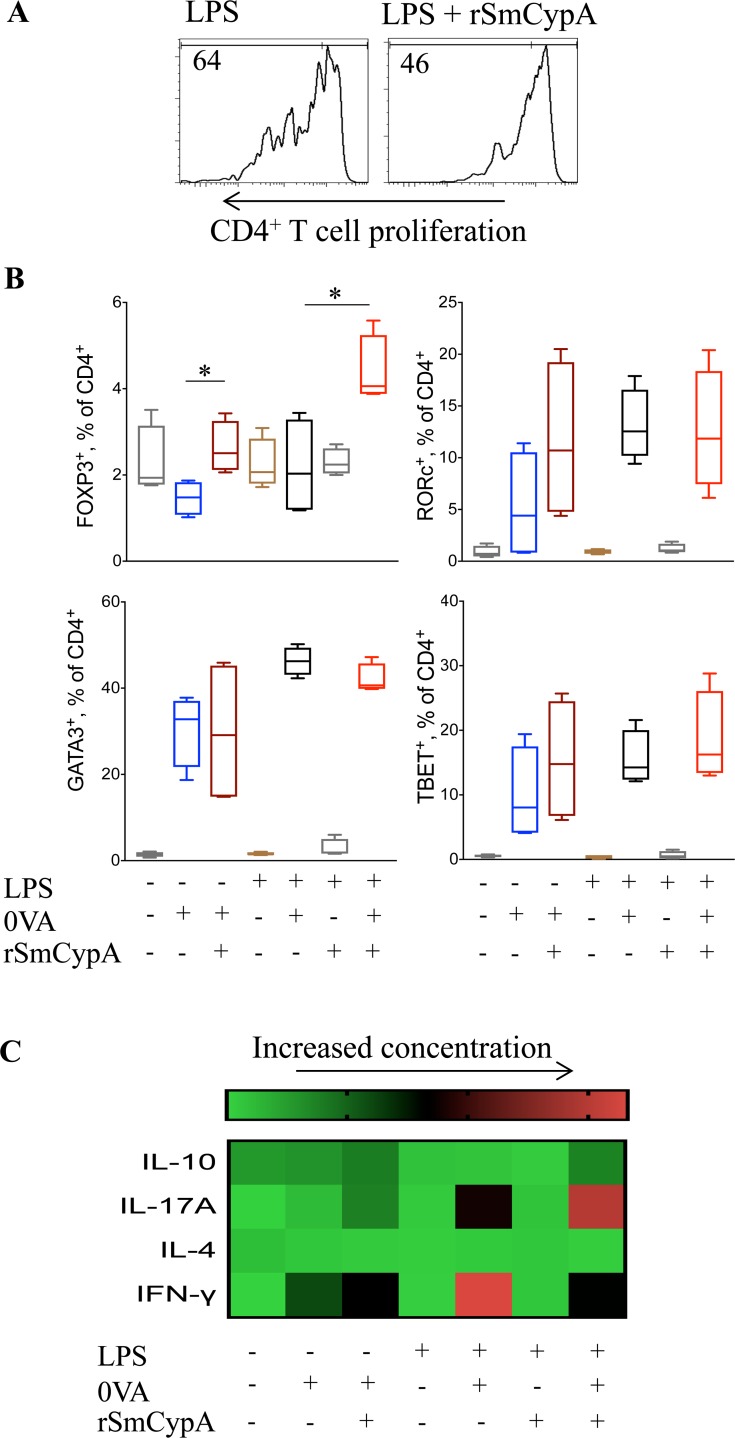
Modulation of CD4^+^ T cells by SmCypA-treated BMDC. (**A**) Representative histograms from flow cytometric analysis of CFSE labeled TCR^OVA^ CD4^+^ T cell cultures with OVA and LPS treated or LPS and rSmCypA co-treated BMDC as APC. (**B**) Flow cytometric analysis for the identification of Treg (FOXP3^+^), Th17 (RORc^+^), Th2 (GATA3^+^) and Th1 (TBET^+^) CD4^+^ T cells following culture with OVA and BMDC as APC, activated under the outlined conditions, n = 4 per group. (**C**) Heatmap of ELISA for the detection of IL-10, IL-17A, IFN-γ and IL-4 in the supernatant of TCR^OVA^ CD4^+^ T cells and BMDC co-cultures. BMDC were activated, or not, under the indicated conditions prior to their co-culture with the TCR^OVA^ CD4^+^ T cells ± antigen (OVA), n = 6 per group. Data are presented as mean and SEM and statistical difference between groups was determined using Student's *t* test.

We next assessed the capacity of BMDC co-treated with LPS and rSmCypA to process and present antigens and subsequently drive T cell activation. CD4^+^ T cells isolated from TCR^OVA^ mice were labeled with CFSE and cultured in the presence of OVA with LPS + rSmCypA co-treated BMDC. rSmCypA treatment of BMDC did not alter the viability of TCR^OVA^ CD4^+^ T cells, but reduced their ability to evoke antigen-specific proliferation of T cells (**Fig 4A**). These results indicate that the reduced CD4^+^ T cell proliferation in response to rSmCypA treated BMDC, could potentially be the effect of altered T cell skewing with a preferential expansion of suppressive Treg cells. BMDC with LPS stimulation and rSmCypA treatment were cultured with TCR^OVA^ CD4^+^ T cells ± antigen (**Fig 4B**). Interestingly, rSmCypA-treated BMDC induced significantly (P<0.05) increased Foxp3^+^ Treg cell expansion but reduced Gata3^+^ Th2, while Rorc^+^ Th17 cell expansion had a modest increase (**Fig 4B**). The rSmCypA expansion of Treg cells was both dose and antigen dependent and it occurs irrespectively of LPS-induced stimulation (**Fig 4B**). In accordance with a preferential expansion of Foxp3^+^ Treg cells, when BMDC co-treated with rSmCypA and LPS were used as APC for the activation of TCR^OVA^ CD4^+^ T cells there is a significant (P<0.05) increase in secreted IL-10 and a decrease (P<0.05) in IFN-γ, marked elevated IL-17A, while IL-4 levels remained unchanged (**Fig 4C**).

## Discussion

*S*. *mansoni* infection can modulate host immunity with the molecules that are excreted or secreted from adult worms acting at the interface of engagement with the immune system of the infected host. The characterisation of WES will potentially be of great significance in the identification of novel therapeutic targets for *S*. *mansoni* and other Schistosoma species’ infections of man [53, 54]. Several previously proposed vaccine candidates were found to be located in extracellular vesicles of adult *S*. *mansoni* worms [55]. In addition to the identification of vaccine candidates, adult *S*. *mansoni* worm proteome has previously been serologically screened for the successful detection of biomarkers and infection susceptibility markers [56]. In our study, 111 proteins of the *S*. *mansoni* male WES were identified by mass-spectometric analysis. This led to the identification of 7 previously proposed vaccine candidates and 5 molecules with potential immunomodulatory activity. Over 70% of the identified *S*. *mansoni* WES proteins share high homology with proteins described in *S*. *japonicum*. Our data show a clear demarcation of the WES and the AW of *S*. *mansoni*, revealing that only a fraction of the *S*. *mansoni* worm proteins are excreted/secreted. It should be noted that WES and AW used in this study were prepared from adult sexually mature male worms, with female worms not included in antigen preparations. This was to remove female worms and therefore presence of eggs, and release of egg secretions during *in vitro* culture, and prevent the potential confounding effects of the potent IM in eggs [57]. Further, infections of mice with male worms only have been shown to induce marked modulation of the immune system of the host [8]. Amongst the WES proteins, an enzymatically active homologue of human CypA was identified. SmCypA showed immunomodulatory activity in *in vitro* cell culture assays, with alterations in DC function and cytokine production leading to a DC mediated preferential expansion of CD4^+^ Treg cells.

Treg cells are important for limiting immunopathology during helminth infections, with significant expansion of these cells during helminth infections [9]. Evidence suggests that this effect is restricted to live helminths, indicating that Treg cell expansion is a result of released components found in the parasite’s WES, with TLR ligands playing a key role in DC modulation and subsequent Treg cell expansion [17, 58]. In this study, we identified SmCypA as a WES component that modulates DC to induce Treg cell expansion *in vitro*. It remains to fully characterise the FoxP3^+^ Treg cells that are expanded by SmCypA treated BMDC *in vitro* and further address the functionality of such cells in mediating the suppression of bystander T cells. Further, in the context of *in vivo* functions of SmCypA, it would be important to determine if modulation of DC by SmCypA induced Treg cells *in vivo*.

Cyclophilins are a group of proteins that have peptidyl-prolyl cis-trans isomerase activity, which have been identified in both prokaryotes and eukaryotes. These proteins are widely expressed and are found in several subcellular compartments at micromolar levels [59]. Cyclophilins have broad functionality, including roles as chaperones and cell signalling molecules [60, 61]. Initially identified as intracellular proteins, later studies showed that CypA and CypB could be secreted under conditions of stress. However, the exact mechanisms and degree of secretion of CypA and CypB are not fully understood [62, 63]. Elucidating these mechanisms would provide a greater understanding of the roles of cyclophilins during inflammation. The first member of the cyclophilins to be identified in mammals, cyclophilin A, is the major cellular target for the immunosuppressive drug cyclosporin A (CsA) as well as newly developed small molecule analogs of CsA that have been shown to limit inflammation and injury by inhibiting neutrophilia in a model of LPS induced acute lung injury [63].

Identification of SmCypA in the adult male worm secretome, highlights the different means by which species can produce similar proteins as part of their immune-suppressive stratagem (as demonstrated by **S2 Table**). Interestingly, a CypA homologue has been identified and shown to be present in all developmental stages of *S*. *japonicum* and in both male and female worms [64]. In *S*.*mansoni* CypA was detected by PCR in both adult male and females worms (**S3 Fig**). Furthermore, *S*.*mansoni*-infected patients have IgE responses against the protein [65], supporting that SmCypA is recognized by the infected host during infection. While these studies highlight the potential importance of schistosome CypA in modulation of the host’s immune system, in the context of human schistosomiasis we have not explored if SmCypA can modulate human DC and T cells.

Several recent studies show that helminths have the ability to alter DC function leading to the generation of DC that can significantly dampen immune responses. Adoptive transfer of BMDC exposed to helminth homogenates, resulted in CD4^+^ T cell IL-10 mediated disease suppression in an experimental model of colitis [66]. Chronic helminth infections have additionally been shown to evoke a preferential expansion of CD11c^lo^ DC that are less capable at inducing T cell proliferation and Th2 cytokine secretion in comparison to CD11c^hi^ DC [67]. Despite recent advances, the mechanisms that lead to alterations in DC function during *S*. *mansoni* infection are not fully understood. While previous studies show that predominantly helminth WES and not AW components are able to modulate DC function and result in the generation of a Th2/Treg skewed response [68, 69], limited individual WES components that induce Treg cells have been described, including a *H*. *polygyrus* TGF-β homologue [70]

We have not explored how rSmCypA interacts with BMDC and thereby modulates their function. Studies by Zhu *et al*., have highlighted that recombinant human CypA binds the CD147 receptor with ligation inducing elevated pERK, pStat3 and pAkt [71]. Whereas, CypA derived from *Histoplasma capsulatum* has been described as binding to the VLA-5 receptor on DC that modifies its adhesion properties [49]. While these studies showed extracellular CypA to have a preferentially pro-inflammatory effect [49, 71], in this study we show that rSmCypA has a DC immune-regulating capacity that attenuates the veracity of DC mediated T cell activation by specifically inducing a Treg cell response. It is possible therefore that rSmCypA competes with the abundant endogenous host CypA during infection to modulate the infected host’s immune system. This highlights that further work is required to elucidate the exact mechanism(s) of action for rSmCypA modulation of DC.

Whilst it is clear that *Schistosoma* species may utilize CypA as an immune modulator, it must be emphasized that this is just one of the potential IM that are produced by the parasite. A number of the other molecules in WES may also have immunomodulatory activity. In addition to this, understanding their composition, relative to each other, is key to elucidate how elegantly helminths modulate immune responses. Further work will be required to carefully delineate the vast array of molecules which helminths generate and to understand how this unique composition of IM act together for immune modulation.

## Supporting information

S1 Fig Isolation and preparation of WES molecules. (**A**) Infected mice were perfused 7 weeks post-infection for collection of adult worms. Males were carefully separated from females and transferred to a dialysis bag containing incubation media, in a cell culture flask with nutrient media. Worms were then incubated for 72 hours and the incubation media harvested. (**B**) Quality control of WES batches was performed by silver staining in three independent batches (B1, B2 and B3 above). (**C**) Western blot of preparations with polyclonal anti-WES rabbit serum (1:1400 dilution) and HRP-conjugated anti-rabbit IgG (1:2000 dilution). Serum collected prior to rabbit (normal rabbit serum—NRS) immunization with WES molecules was used as a negative control.
(TIFF)

S2 Fig Gating strategy for flow cytometric analysis of BMDC. **Representative e**xample of the gating strategy followed for the flow cytometry analysis of BMDC and following activation of BMDC with LPS.
(TIFF)

S3 Fig *SmCypa* expression by adult male and female *S*. *mansoni* worms. **(A)** Representative images of agarose gel electrophoresis of PCR products for expression of *SmCypa* (mRNA) and housekeeping genes *Pai1* (mRNA) and *Gapdh* (mRNA) by male or female adult *S*. *mansoni* worms. **(B)** Fold expression change of *SmCypa* mRNA for male only and female only *S*. *mansoni* adult worms, compared to housekeeping genes *Pai1* or *Gapdh*, *n = 3*, data are presented as mean and SEM.
(TIFF)

S1 Table *S*. *mansoni* adult male worm excretory-secretory proteins. ID–identification number at GeneDB; MS–mowse score; CO*—*percentage of sequence coverage; SP–signal peptide; SecP–SecretomeP score, values above 0.5 indicate possible secretion; TM*—*number of transmembrane domains; (+)*—*signal peptide detected; (-) no signal peptide or transmembrane domain detected.
(DOCX)

S2 Table *S*. *mansoni* adult male worm excretory-secretory protein homologues. *S*. *mansoni* WES proteins were subjected to similarity analysis using BLAST. Sequence alignments that showed a bit score higher than 30 and an E value lower than 1e-16 were considered homologs. ID–identification number at GeneDB or GenBank databases; (-)–no homologs found.
(DOCX)
