# Supplementary figures and images for: Composition of the Schistosoma mansoni worm secretome: Identification of immune modulatory Cyclophilin A
Source: PLoS Negl Trop Dis. 2017 Oct 26;11(10):e0006012. doi: 10.1371/journal.pntd.0006012 (PMC5681295; doi:10.1371/journal.pntd.0006012)

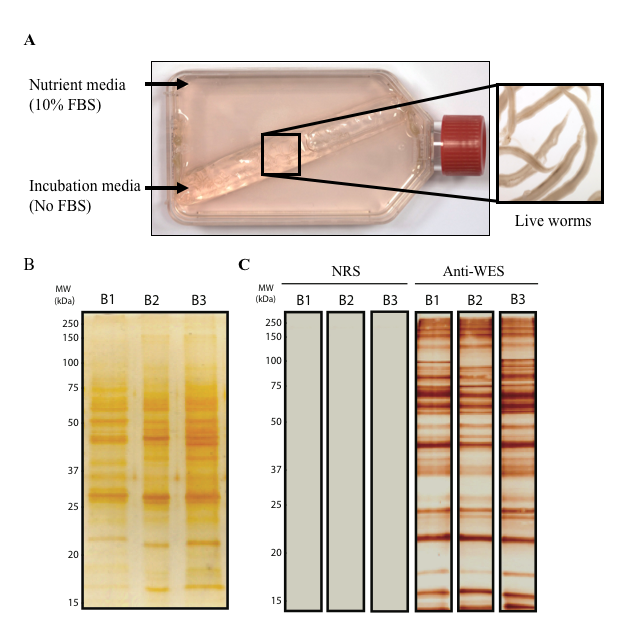

Supplement: S1 Fig — (A) Infected mice were perfused 7 weeks post-infection for collection of adult worms. Males were carefully separated from females and transferred to a dialysis bag containing incubation media, in a cell culture flask with nutrient media. Worms were then incubated for 72 hours and the incubation media harvested. (B) Quality control of WES batches was performed by silver staining in three independent batches (B1, B2 and B3 above). (C) Western blot of preparations with polyclonal anti-WES rabbit serum (1:1400 dilution) and HRP-conjugated anti-rabbit IgG (1:2000 dilution). Serum collected prior to rabbit (normal rabbit serum—NRS) immunization with WES molecules was used as a negative control. (TIFF) [file pntd.0006012.s001.tiff]

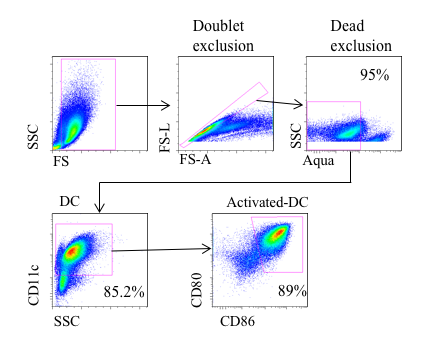

Supplement: S2 Fig — Representative example of the gating strategy followed for the flow cytometry analysis of BMDC and following activation of BMDC with LPS. (TIFF) [file pntd.0006012.s002.tiff]

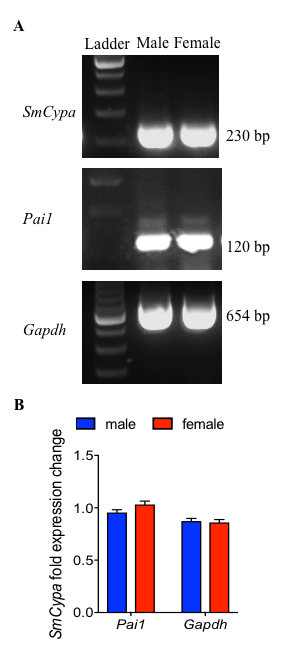

Supplement: S3 Fig — (A) Representative images of agarose gel electrophoresis of PCR products for expression of SmCypa (mRNA) and housekeeping genes Pai1 (mRNA) and Gapdh (mRNA) by male or female adult S. mansoni worms. (B) Fold expression change of SmCypa mRNA for male only and female only S. mansoni adult worms, compared to housekeeping genes Pai1 or Gapdh, n = 3, data are presented as mean and SEM. (TIFF) [file pntd.0006012.s003.tiff]
