# Supplementary material for: Composition of the Schistosoma mansoni worm secretome: Identification of immune modulatory Cyclophilin A
Source: PLoS Negl Trop Dis. 2017 Oct 26;11(10):e0006012. doi: 10.1371/journal.pntd.0006012 (PMC5681295; doi:10.1371/journal.pntd.0006012)
Supplement: S1 Table — ID–identification number at GeneDB; MS–mowse score; CO—percentage of sequence coverage; SP–signal peptide; SecP–SecretomeP score, values above 0.5 indicate possible secretion; TM—number of transmembrane domains; (+)—signal peptide detected; (-) no signal peptide or transmembrane domain detected. (DOCX) [file pntd.0006012.s004.docx]

| **ID** | | **Protein description** | **MS** | **CO** | **SP** | **SecP** | **TM** |
| --- | --- | --- | --- | --- | --- | --- | --- |
| 1 | Smp_000100 | Filamin | 467 | 5% | - | 0.539 | - |
| 2 | Smp_000660 | Ornithine--oxo-acid transaminase | 444 | 25% | - | 0.424 | - |
| 3 | Smp_001360 | Thymidylate kinase | 596 | 57% | - | 0.747 | - |
| 4 | Smp_003230 | Sh3 domain grb2-like protein B1 (endophilin B1) | 218 | 31% | - | 0.801 | - |
| 5 | Smp_003990 | Triosephosphate isomerase, putative | 1048 | 56% | - | 0.508 | - |
| 6 | Smp_004350 | Ubiquitin-conjugating enzyme E2 l, putative | 74 | 10% | - | 0.681 | - |
| 7 | Smp_004470.1 | Peroxiredoxin, Prx3 | 136 | 20% | - | 0.672 | - |
| 8 | Smp_004780.1 | Immunophilin, putative | 398 | 24% | - | 0.27 | - |
| 9 | Smp_005350 | Calcium-binding protein, putative | 512 | 16% | - | 0.834 | - |
| 10 | Smp_006390 | Cystatin B, putative | 421 | 58% | - | 0.562 | - |
| 11 | Smp_007270.1 | Alpha-actinin, putative | 193 | 24% | - | 0.488 | - |
| 12 | Smp_008070 | Thioredoxin, Trx1 | 551 | 79% | - | 0.585 | - |
| 13 | Smp_008110 | WD40-repeat containing protein | 592 | 13% | - | 0.558 | - |
| 14 | Smp_008660.1 | Gelsolin, putative | 1238 | 62% | - | 0.406 | - |
| 15 | Smp_009760 | 14-3-3 protein, putative | 416 | 33% | - | 0.246 | - |
| 16 | Smp_009780.2 | 14-3-3 protein, putative | 245 | 19% | - | 0.414 | - |
| 17 | Smp_011830 | Hypothetical protein / C4Q068 | 118 | 21% | - | 0.845 | - |
| 18 | Smp_014010 | Adenylyl cyclase-associated protein, putative | 177 | 11% | - | 0.548 | - |
| 19 | Smp_017730 | 200-kDa GPI-anchored surface glycoprotein | 134 | 2% | + | 0.667 | - |
| 20 | Smp_018240.3 | Cell division control protein 48 aaa family protein, putative | 339 | 12% | - | 0.179 | - |
| 21 | Smp_018890 | Phosphoglycerate kinase | 901 | 78% | - | 0.442 | - |
| 22 | Smp_019050.2 | Hypothetical protein / C4Q286 | 367 | 32% | - | 0.728 | - |
| 23 | Smp_019640.1 | Calcyphosine/tpp, putative | 213 | 26% | - | 0.516 | - |

| **ID** | | **Protein description** | **MS** | **CO** | **SP** | **SecP** | **TM** |
| --- | --- | --- | --- | --- | --- | --- | --- |
| 24 | Smp_020920.1 | DNAj homolog subfamily B member 4, putative | 608 | 35% | - | 0.661 | - |
| 25 | Smp_021800 | Prefoldin subunit 3-related | 358 | 37% | - | 0.49 | - |
| 26 | Smp_022340 | Pdz and lim domain protein, putative | 225 | 14% | - | 0.609 | - |
| 27 | Smp_024110 | Phosphopyruvate hydratase | 1214 | 51% | - | 0.471 | - |
| 28 | Smp_028670.1 | Carbonic anhydrase II (carbonate dehydratase II), putative | 265 | 22% | - | 0.809 | - |
| 29 | Smp_030000 | Leucine aminopeptidase (M17 family) | 377 | 23% | - | 0.545 | - |
| 30 | Smp_030370 | Calreticulin autoantigen homolog precursor, putative | 1292 | 53% | + | 0.504 | - |
| 31 | Smp_030730 | Tubulin beta chain, putative | 1074 | 47% | - | 0.509 | - |
| 32 | Smp_031770.4 | Tropomyosin, putative | 2078 | 90% | - | 0.454 | - |
| 33 | Smp_032580.2 | Subfamily T1A non-peptidase homologue (T01 family) | 572 | 43% | - | 0.372 | - |
| 34 | Smp_032950 | Calmodulin (CaM), putative | 197 | 44% | - | 0.742 | - |
| 35 | Smp_033540 | Carbonyl reductase, putative | 131 | 14% | - | 0.387 | - |
| 36 | Smp_034490 | Proteasome catalytic subunit 1 (T01 family) | 238 | 26% | - | 0.435 | - |
| 37 | Smp_034840.2 | 14-3-3 epsilon | 288 | 25% | - | 0.225 | - |
| 38 | Smp_035270.2 | Malate dehydrogenase, putative | 404 | 27% | - | 0.343 | - |
| 39 | Smp_038950 | L-lactate dehydrogenase, putative | 337 | 19% | - | 0.492 | - |
| 40 | Smp_040130 | Cyclophilin | 927 | 77% | - | 0.543 | - |
| 41 | Smp_040790 | Cyclophilin B, putative | 113 | 19% | + | 0.884 | - |
| 42 | Smp_042160.2 | Fructose 1,6-bisphosphate aldolase, putative | 1775 | 72% | - | 0.317 | - |
| 43 | Smp_042400 | Hypothetical protein / C4Q8L5 | 161 | 25% | + | 0.739 | - |
| 44 | Smp_043030 | Hexokinase | 121 | 9% | - | 0.283 | - |
| 45 | Smp_043120 | Universal stress protein, putative | 69 | 9% | - | 0.536 | - |
| 46 | Smp_044010.2 | Tropomyosin, putative | 1230 | 60% | - | 0.38 | - |

| **ID** | | **Protein description** | **MS** | **CO** | **SP** | **SecP** | **TM** |
| --- | --- | --- | --- | --- | --- | --- | --- |
| 47 | Smp_046600 | Actin-1, putative | 1188 | 49% | - | 0.5 | - |
| 48 | Smp_046690 | Ubiquitin (ribosomal protein L40), putative | 185 | 16% | - | 0.411 | - |
| 49 | Smp_047370 | Malate dehydrogenase, putative | 395 | 19% | - | 0.5 | - |
| 50 | Smp_047650 | Ferritin, putative | 455 | 57% | - | 0.676 | - |
| 51 | Smp_049250 | Heat shock protein, putative | 333 | 22% | - | 0.823 | 1 |
| 52 | Smp_049270 | Heat shock protein, putative | 139 | 7% | - | 0.684 | - |
| 53 | Smp_049550 | Heat shock protein 70 (hsp70), putative | 163 | 6% | + | 0.546 | - |
| 54 | Smp_050390 | Aldehyde dehydrogenase, putative | 814 | 36% | - | 0.535 | - |
| 55 | Smp_053220.1 | Aldo-keto reductase, putative | 759 | 46% | - | 0.393 | - |
| 56 | Smp_054160 | Glutathione S-transferase 28 kDa (GST 28) (GST class-mu), putative | 1338 | 90% | - | 0.3 | - |
| 57 | Smp_054240 | Translationally-controlled tumor protein homolog (TCTP) (Histamine-releasing factor), putative | 583 | 59% | - | 0.407 | - |
| 58 | Smp_056440 | Superoxide dismutase [mn], putative | 88 | 10% | - | 0.689 | - |
| 59 | Smp_056760 | Protein disulfide-isomerase, putative | 1340 | 54% | + | 0.805 | - |
| 60 | Smp_056970.1 | Glyceraldehyde-3-phosphate dehydrogenase (phosphorylating) | 966 | 47% | - | 0.412 | - |
| 61 | Smp_059480 | Peroxiredoxin, Prx1 | 450 | 49% | - | 0.597 | - |
| 62 | Smp_059660 | Hypothetical protein / C4QDG6 | 67 | 4% | - | 0.533 | - |
| 63 | Smp_059980 | Arginase, putative | 307 | 22% | - | 0.493 | - |
| 64 | Smp_063120.1 | Inosine triphosphate pyrophosphatase (itpase) (inosine triphosphatase), putative | 229 | 42% | - | 0.571 | - |
| 65 | Smp_063530.1 | Apoferritin-2 | 593 | 51% | + | 0.711 | - |
| 66 | Smp_064380 | Aspartate aminotransferase, putative | 231 | 13% | - | 0.544 | - |
| 67 | Smp_064860 | Heat shock protein 70 (hsp70)-interacting protein, putative | 517 | 36% | - | 0.448 | - |
| 68 | Smp_066760.2 | Merlin/moesin/ezrin/radixin, putative | 102 | 5% | - | 0.328 | - |

| **ID** | | **Protein description** | **MS** | **CO** | **SP** | **SecP** | **TM** |
| --- | --- | --- | --- | --- | --- | --- | --- |
| 69 | Smp_067890 | Proteasome subunit alpha 2 (T01 family) | 588 | 37% | - | 0.443 | - |
| 70 | Smp_072900.1 | Hsp90 co-chaperone (tebp), putative | 77 | 8% | - | 0.344 | - |
| 71 | Smp_078690 | Calponin homolog, putative | 570 | 49% | - | 0.575 | - |
| 72 | Smp_079010 | Camp-dependent protein kinase type II-alpha regulatory subunit, putative | 261 | 21% | - | 0.596 | - |
| 73 | Smp_079770.1 | Protein disulfide-isomerase ER-60 precursor (ERP60), putative | 999 | 41% | + | 0.662 | - |
| 74 | Smp_081430 | Short chain dehydrogenase, putative | 170 | 22% | - | 0.28 | - |
| 75 | Smp_082030 | Family C56 non-peptidase homologue (C56 family) | 386 | 54% | - | 0.498 | - |
| 76 | Smp_083870 | PwLAP aminopeptidase (M17 family) | 193 | 10% | - | 0.449 | - |
| 77 | Smp_086330.2 | Calponin-related | 260 | 32% | - | 0.732 | - |
| 78 | Smp_086480 | Antigen Sm21.7, putative | 222 | 25% | - | 0.658 | - |
| 79 | Smp_086530 | Tegumental protein Sm 20.8, putative | 250 | 29% | - | 0.36 | - |
| 80 | Smp_090080 | Serpin, putative | 806 | 36% | - | 0.601 | - |
| 81 | Smp_090120.1 | Alpha tubulin, putative | 68 | 3% | - | 0.475 | - |
| 82 | Smp_091010 | Glyoxalase II (Hydroxyacylglutathione hydrolase), putative | 568 | 42% | - | 0.386 | - |
| 83 | Smp_092280 | Proteasome subunit alpha 3 (T01 family) | 724 | 45% | - | 0.384 | - |
| 84 | Smp_092750 | Nucleoside diphosphate kinase | 329 | 63% | - | 0.384 | - |
| 85 | Smp_095360.1 | Fatty acid binding protein | 325 | 54% | - | 0.798 | - |
| 86 | Smp_096760 | Phosphoglycerate mutase | 419 | 34% | - | 0.293 | - |
| 87 | Smp_102070 | GST class-mu, SM26/2 antigen, glutathione S-transferase 26 kDa | 878 | 60% | - | 0.445 | - |
| 88 | Smp_103320 | Nuclear movement protein nudc, putative | 193 | 7% | - | 0.345 | - |
| 89 | Smp_105020 | Titin, putative | 66 | 4% | - | 0.571 | - |
| 90 | Smp_106930.2 | Heat shock protein 70, putative | 1324 | 46% | - | 0.273 | - |
| 91 | Smp_123440.1 | Fad oxidoreductase, putative | 123 | 12% | - | 0.551 | - |

| **ID** | | **Protein description** | **MS** | **CO** | **SP** | **SecP** | **TM** |
| --- | --- | --- | --- | --- | --- | --- | --- |
| 92 | Smp_130110 | Proteasome subunit alpha 6 (T01 family) | 691 | 18% | - | 0.471 | - |
| 93 | Smp_132670.1 | Myosin regulatory light chain, putative | 162 | 16% | - | 0.567 | - |
| 94 | Smp_135950 | Lethal giant larvae homolog 2, cell polarity protein , inorganic pyrophosphatase, putative | 316 | 5% | - | 0.476 | - |
| 95 | Smp_136240.6 | Vesicle-associated membrane protein (vamp), putative | 161 | 10% | - | 0.556 | 1 |
| 96 | Smp_140900.2 | Hypothetical protein / C4Q6S1 | 156 | 19% | - | 0.512 | - |
| 97 | Smp_143470.2 | Spectrin beta chain, brain 3 (Spectrin, non- erythroid beta chain 3) (Beta-IV spectrin), putative | 100 | 1% | - | 0.229 | - |
| 98 | Smp_146950 | Hypothetical protein / C4Q9Q0 | 286 | 1% | - |  | - |
| 99 | Smp_147470 | Leucine-rich transmembrane proteins, putative | 116 | 7% | - | 0.064 | 1 |
| 100 | Smp_150820 | Acyl-CoA thioesterase-related | 174 | 7% | - | 0.867 | - |
| 101 | Smp_151690 | Translation initiation inhibitor, putative | 205 | 42% | - | 0.649 | - |
| 102 | Smp_152710.2 | Glutathione-s-transferase omega, putative | 941 | 63% | - | 0.418 | - |
| 103 | Smp_155060.2 | Set, putative | 201 | 14% | - | 0.424 | - |
| 104 | Smp_157500 | Calpain (C02 family) | 393 | 5% | - | 0.406 | - |
| 105 | Smp_158110.2 | Peroxiredoxin, Prx2 | 59 | 9% | - | 0.731 | - |
| 106 | Smp_161920 | Actin, putative | 1114 | 43% | - | 0.511 | - |
| 107 | Smp_163720 | Endophilin B1, putative | 106 | 14% | - | 0.699 | - |
| 108 | Smp_176200.2 | Superoxide dismutase [Cu-Zn] | 372 | 58% | - | 0.569 | - |
| 109 | Smp_179810 | Troponin t, invertebrate, putative | 361 | 20% | - | 0.2 | - |
| 110 | Smp_187370 | Phosphoglycerate kinase | 805 | 70% | - | 0.352 | - |
| 111 | Smp_194770 | ATP:guanidino kinase (Smc74), putative | 834 | 23% | - | 0.402 | - |

**Table S1: *S. mansoni* adult male worm excretory-secretory proteins.** ID – identification number at GeneDB; MS – mowse score; CO *-* percentage of sequence coverage; SP – signal peptide; SecP – SecretomeP score, values above 0.5 indicate possible secretion; TM *-* number of transmembrane domains; (+) *-* signal peptide detected; (-) no signal peptide or transmembrane domain detected.
