# Supplementary material for: Composition of the Schistosoma mansoni worm secretome: Identification of immune modulatory Cyclophilin A
Source: PLoS Negl Trop Dis. 2017 Oct 26;11(10):e0006012. doi: 10.1371/journal.pntd.0006012 (PMC5681295; doi:10.1371/journal.pntd.0006012)
Supplement: S2 Table — S. mansoni WES proteins were subjected to similarity analysis using BLAST. Sequence alignments that showed a bit score higher than 30 and an E value lower than 1e-16 were considered homologs. ID–identification number at GeneDB or GenBank databases; (-)–no homologs found. (DOCX) [file pntd.0006012.s005.docx]

| ***S. mansoni* male adult worm** | | | ***S. japonicum*** | | ***S. haematobium*** | |
| --- | --- | --- | --- | --- | --- | --- |
| **excretory-secretory proteins** | | |  |  |  |  |
| **GeneDB ID** | | **Protein description** | **GenBank ID** | **Identity (%)** | **GenBank ID** | **Identity (%)** |
| 1 | Smp_000100 | Filamin | AAF13300.1 | 50.5 | - | - |
| 2 | Smp_000660 | Ornithine--oxo-acid transaminase | CAX70598.1 | 9252 | - | - |
| 3 | Smp_001360 | Thymidylate kinase | CAX75696.1 | 84.82 | - | - |
| 4 | Smp_003230 | Sh3 domain grb2-like protein B1 (endophilin B1) | CAX70480.1 | 68.15 | - | - |
| 5 | Smp_003990 | Triosephosphate isomerase, putative | AAP06170.1 | 92.86 | BAF62292.1 | 95.24 |
| 6 | Smp_004350 | Ubiquitin-conjugating enzyme E2 l, putative | AAW27854.1 | 98.08 | - | - |
| 7 | Smp_004470.1 | Peroxiredoxin, Prx3 | AAW25436.1 | 88.18 | - | - |
| 8 | Smp_004780.1 | Immunophilin, putative | AAW27121.1 | 78.25 | - | - |
| 9 | Smp_005350 | Calcium-binding protein, putative | AAP06154.1 | 100 | - | - |
| 10 | Smp_006390 | Cystatin B, putative | CAX73577.1 | 77.23 | - | - |
| 11 | Smp_007270.1 | Alpha-actinin, putative | CAX82586.1 | 93.75 | - | - |
| 12 | Smp_008070 | Thioredoxin, Trx1 | AAD52699.1 | 65.38 | - | - |
| 13 | Smp_008110 | WD40-repeat containing protein | - | - | - | - |
| 14 | Smp_008660.1 | Gelsolin, putative | AAW26119.1 | 83.1 | - | - |
| 15 | Smp_009760 | 14-3-3 protein, putative | ACE06842.1 | 69.05 | - | - |
| 16 | Smp_009780.2 | 14-3-3 protein, putative | ACE06842.1 | 67.23 | - | - |
| 17 | Smp_011830 | Hypothetical protein / C4Q068 | AAW26653.1 | 85.58 | - | - |
| 18 | Smp_014010 | Adenylyl cyclase-associated protein, putative | CAX69899.1 | 75.83 | - | - |
| 19 | Smp_017730 | 200-kDa GPI-anchored surface glycoprotein | AAX26034.2 | 72.44 | - | - |
| 20 | Smp_018240.3 | Cell division control protein 48 aaa family protein, putative | AAW27581.1 | 92.28 | - | - |
| 21 | Smp_018890 | Phosphoglycerate kinase | AAP06480.1 | 94.24 | - | - |
| 22 | Smp_019050.2 | Hypothetical protein / C4Q286 | CAX73085.1 | 80.37 | - | - |

| ***S. mansoni* male adult worm** | | | ***S. japonicum*** | | ***S. haematobium*** | |
| --- | --- | --- | --- | --- | --- | --- |
| **excretory-secretory proteins** | | |  |  |  |  |
| **GeneDB ID** | | **Protein description** | **GenBank ID** | **Identity (%)** | **GenBank ID** | **Identity (%)** |
| 23 | Smp_019640.1 | Calcyphosine/tpp, putative | AAW27463.1 | 83.17 | - | - |
| 24 | Smp_020920.1 | DNAj homolog subfamily B member 4, putative | AAW25539.1 | 91.94 | AAD00565.1 | 45.45 |
| 25 | Smp_021800 | Prefoldin subunit 3-related | AAW27184.1 | 90.48 | - | - |
| 26 | Smp_022340 | Pdz and lim domain protein, putative | AAW27396.1 | 84.26 | - | - |
| 27 | Smp_024110 | Phosphopyruvate hydratase | P33676.1 | 87.79 | - | - |
| 28 | Smp_028670.1 | Carbonic anhydrase II (carbonate dehydratase II), putative | CAX73485.1 | 93 | - | - |
| 29 | Smp_030000 | Leucine aminopeptidase (M17 family) | CAX69903.1 | 88.85 | - | - |
| 30 | Smp_030370 | Calreticulin autoantigen homolog precursor, putative | AAC00515.1 | 77.27 | - | - |
| 31 | Smp_030730 | Tubulin beta chain, putative | CAX71985.1 | 99.32 | - | - |
| 32 | Smp_031770.4 | Tropomyosin, putative | CAX76350.1 | 98.94 | Q26503.1 | 65.02 |
| 33 | Smp_032580.2 | Subfamily T1A non-peptidase homologue (T01 family) | AAP06025.1 | 97.15 | - | - |
| 34 | Smp_032950 | Calmodulin (CaM), putative | CAX79767.1 | 76.47 | - | - |
| 35 | Smp_033540 | Carbonyl reductase, putative | CAX77260.1 | 85.56 | - | - |
| 36 | Smp_034490 | Proteasome catalytic subunit 1 (T01 family) | CAX69953.1 | 89.78 | - | - |
| 37 | Smp_034840.2 | 14-3-3 epsilon | AAW26747.1 | 81.51 | - | - |
| 38 | Smp_035270.2 | Malate dehydrogenase, putative | CAX72207.1 | 87.8 | - | - |
| 39 | Smp_038950 | L-lactate dehydrogenase, putative | CAX70888.1 | 87.65 | - | - |
| 40 | Smp_040130 | Cyclophilin | CAX72371.1 | 69.18 | - | - |
| 41 | Smp_040790 | Cyclophilin B, putative | AAW27862.1 | 84.98 | - | - |
| 42 | Smp_042160.2 | Fructose 1,6-bisphosphate aldolase, putative | AAW25258.1 | 96.14 | - | - |
| 43 | Smp_042400 | Hypothetical protein / C4Q8L5 | AAW24701.1 | 79.01 | - | - |
| 44 | Smp_043030 | Hexokinase | CAX69908.1 | 88.22 | - | - |

| ***S. mansoni* male adult worm** | | | | | ***S. japonicum*** | | | ***S. haematobium*** | | |
| --- | --- | --- | --- | --- | --- | --- | --- | --- | --- | --- |
| **excretory-secretory proteins** | | | | |  |  |  |  |  |  |
| **GeneDB ID** | | | **Protein description** | | **GenBank ID** | **Identity (%)** | | **GenBank ID** | **Identity (%)** | |
| 45 | | Smp_043120 | | Universal stress protein, putative | CAX70901.1 | | 80.62 | - | | - |
| 46 | | Smp_044010.2 | | Tropomyosin, putative | ACE06925.1 | | 98.24 | - | | - |
| 47 | Smp_046600 | | Actin-1, putative | | CAX69775.1 | 98.14 | | - | - | |
| 48 | Smp_046690 | | Ubiquitin (ribosomal protein L40), putative | | CAX72429.1 | 99.67 | | - | - | |
| 49 | Smp_047370 | | Malate dehydrogenase, putative | | CAX74903.1 | 93.84 | | - | - | |
| 50 | Smp_047650 | | Ferritin, putative | | CAX70640.1 | 87.21 | | - | - | |
| 51 | Smp_049250 | | Heat shock protein, putative | | CAX78232.1 | 62.73 | | - | - | |
| 52 | Smp_049270 | | Heat shock protein, putative | | AAW24545.1 | 90.36 | | - | - | |
| 53 | Smp_049550 | | Heat shock protein 70 (hsp70), putative | | ACE06854.1 | 91.05 | | - | - | |
| 54 | Smp_050390 | | Aldehyde dehydrogenase, putative | | CAX73522.1 | 90.02 | | - | - | |
| 55 | Smp_053220.1 | | Aldo-keto reductase, putative | | CAX77280.1 | 83.87 | | - | - | |
| 56 | Smp_054160 | | Glutathione S-transferase 28 kDa (GST 28) (GST class-mu), putative | | CAX72408.1 | 77.25 | | P30114.1 | 91.94 | |
| 57 | Smp_054240 | | Translationally-controlled tumor protein homolog (TCTP) (Histamine-releasing factor), putative | | P91800.1 | 58.82 | | Q8I8A2.2 | 80.29 | |
| 58 | Smp_056440 | | Superoxide dismutase [mn], putative | | AAW26480.1 | 89.81 | | - | - | |
| 59 | Smp_056760 | | Protein disulfide-isomerase, putative | | CAX69780.1 | 89.83 | | - | - | |
| 60 | Smp_056970.1 | | Glyceraldehyde-3-phosphate dehydrogenase (phosphorylating) | | CAX80263.1 | 90.24 | | - | - | |
| 61 | Smp_059480 | | Peroxiredoxin, Prx1 | | CAX71944.1 | 83.7 | | - | - | |
| 62 | Smp_059660 | | Hypothetical protein / C4QDG6 | | AAP06314.1 | 77.93 | | - | - | |
| 63 | Smp_059980 | | Arginase, putative | | CAX70201.1 | 80.22 | | - | - | |
| 64 | Smp_063120.1 | | Inosine triphosphate pyrophosphatase (itpase) (inosine triphosphatase), putative | | AAX27755.2 | 84.78 | | - | - | |
| 65 | Smp_063530.1 | | Apoferritin-2 | | CAX72682.1 | 84.46 | | - | - | |

Table 3.2 (continued)

| ***S. mansoni* male adult worm** | | | | ***S. japonicum*** | | | ***S. haematobium*** | | | |
| --- | --- | --- | --- | --- | --- | --- | --- | --- | --- | --- |
| **excretory-secretory proteins** | | | |  |  |  |  |  |  |  |
| **GeneDB ID** | | **Protein description** | | **GenBank ID** | **Identity (%)** | | **GenBank ID** | | | **Identity (%)** |
| 66 | Smp_064380 | | Aspartate aminotransferase, putative | CAX69569.1 | | 81.29 | | - | - | |
| 67 | Smp_064860 | | Heat shock protein 70 (hsp70)-interacting protein, putative | AAW27834.1 | | 84.64 | | - | - | |
| 68 | Smp_066760.2 | | Merlin/moesin/ezrin/radixin, putative | CAX82442.1 | | 70.44 | | - | - | |
| 69 | Smp_067890 | Proteasome subunit alpha 2 (T01 family) | | gAAW25457.1 | 96.6 | | - | | | - |
| 70 | Smp_072900.1 | Hsp90 co-chaperone (tebp), putative | | CAX79556.1 | 80.43 | | - | | | - |
| 71 | Smp_078690 | Calponin homolog, putative | | ACE06952.1 | 92.8 | | - | | | - |
| 72 | Smp_079010 | Camp-dependent protein kinase type II-alpha regulatory subunit, putative | | AAW24538.1 | 89.92 | | - | | | - |
| 73 | Smp_079770.1 | Protein disulfide-isomerase ER-60 precursor (ERP60), putative | | ACE06849.1 | 76.08 | | - | | | - |
| 74 | Smp_081430 | Short chain dehydrogenase, putative | | AAW27200.1 | 90.08 | | - | | | - |
| 75 | Smp_082030 | Family C56 non-peptidase homologue (C56 family) | | CAX70856.1 | 86.41 | | - | | | - |
| 76 | Smp_083870 | PwLAP aminopeptidase (M17 family) | | AAX27247.2 | 90.56 | | - | | | - |
| 77 | Smp_086330.2 | Calponin-related | | AAP06498.1 | 95.26 | | - | | | - |
| 78 | Smp_086480 | Antigen Sm21.7, putative | | CAX72713.1 | 64.86 | | AAW49250.1 | | | 47.8 |
| 79 | Smp_086530 | Tegumental protein Sm 20.8, putative | | AAP06272.1 | 78.89 | | BAF62289.1 | | | 30.39 |
| 80 | Smp_090080 | Serpin, putative | | CAX76359.1 | 65.1 | | - | | | - |
| 81 | Smp_090120.1 | Alpha tubulin, putative | | XP_002580033.1 | 100 | | AAW66672.1 | | | 40.32 |
| 82 | Smp_091010 | Glyoxalase II (Hydroxyacylglutathione hydrolase), putative | | AAP06491.1 | 88.51 | | - | | | - |
| 83 | Smp_092280 | Proteasome subunit alpha 3 (T01 family) | | CAX70764.1 | 95.67 | | - | | | - |
| 84 | Smp_092750 | Nucleoside diphosphate kinase | | AAO59410.1 | 88.59 | | - | | | - |
| 85 | Smp_095360.1 | Fatty acid binding protein | | AAP14675.1 | 92.42 | | BAF62288.1 | | | 99.24 |

Table 3.2 (continued)

| ***S. masoni* male adult worm** | | | | | ***S. japonicum*** | | | | ***S. haematobium*** | | | |
| --- | --- | --- | --- | --- | --- | --- | --- | --- | --- | --- | --- | --- |
| **excretory-secretory proteins** | | | | |  |  |  |  |  |  |  |  |
| **GeneDB ID** | | | **Protein description** | | **GenBank ID** | | **Identity (%)** | | **GenBank ID** | | **Identity (%)** | |
| 86 | Smp_096760 | | | Phosphoglycerate mutase | | CAX76329.1 | | 94 | | - | | - |
| 87 | Smp_102070 | | | GST class-mu, SM26/2 antigen, glutathione S-transferase 26 kDa | | P08515.3 | | 82.57 | | - | | - |
| 88 | Smp_103320 | | | Nuclear movement protein nudc, putative | | AAP06040.1 | | 82.98 | | - | | - |
| 89 | Smp_105020 | | | Titin, putative | | AAW24500.1 | | 91.97 | | - | | - |
| 90 | Smp_106930.2 | | | Heat shock protein 70, putative | | AAC00519.1 | | 61.99 | | - | | - |
| 91 | Smp_123440.1 | | | Fad oxidoreductase, putative | | AAW26635.1 | | 83.71 | | - | | - |
| 92 | | Smp_130110 | Proteasome subunit alpha 6 (T01 family) | | AAW25684.1 | | 93.99 | | - | | - | |
| 93 | | Smp_132670.1 | Myosin regulatory light chain, putative | | AAW26951.1 | | 97.14 | | - | | - | |
| 94 | | Smp_135950 | Lethal giant larvae homolog 2, cell polarity protein, inorganic pyrophosphatase, putative | | AW25943.1 | | 87.02 | | - | | - | |
| 95 | | Smp_136240.6 | Vesicle-associated membrane protein (vamp), putative | | AAW26025.1 | | 79.61 | | - | | - | |
| 96 | | Smp_140900.2 | Hypothetical protein / C4Q6S1 | | AAW26313.1 | | 72.65 | | - | | - | |
| 97 | | Smp_143470.2 | Spectrin beta chain, brain 3 (Spectrin, non- erythroid beta chain 3) (Beta-IV spectrin), putative | | AAX26038.2 | | 93.33 | | - | | - | |
| 98 | | Smp_146950 | Hypothetical protein / C4Q9Q0 | | AAX26800.2 | | 62.64 | | - | | - | |
| 99 | | Smp_147470 | Leucine-rich transmembrane proteins, putative | | AAW25815.1 | | 86.61 | | - | | - | |
| 100 | | Smp_150820 | Acyl-CoA thioesterase-related | | AAW27278.1 | | 71.14 | | - | | - | |
| 101 | | Smp_151690 | Translation initiation inhibitor, putative | | CAX74647.1 | | 78.76 | | - | | - | |
| 102 | | Smp_152710.2 | Glutathione-s-transferase omega, putative | | CAX74405.1 | | 75.1 | | - | | - | |
| 103 | | Smp_155060.2 | Set, putative | | CAX72508.1 | | 97.82 | | - | | - | |
| 104 | | Smp_157500 | Calpain (C02 family) | | BAA74718.1 | | 83.91 | | - | | - | |
| 105 | | Smp_158110.2 | Peroxiredoxin, Prx2 | | BAD90102.1 | | 89.69 | | - | | - | |
| 106 | | Smp_161920 | Actin, putative | | XP_002578518.1 | | 100 | | - | | - | |
| 107 | | Smp_163720 | Endophilin B1, putative | | AAP06059.1 | | 64.31 | | - | | - | |
| 108 | | Smp_176200.2 | Superoxide dismutase [Cu-Zn] | | CAX76410.1 | | 85.62 | | - | | - | |
| 109 | | Smp_179810 | Troponin t, invertebrate, putative | | AAW25147.1 | | 92.12 | | - | | - | |
| 110 | | Smp_187370 | Phosphoglycerate kinase | | CAX77845.1 | | 94.77 | | - | | - | |
| 111 | | Smp_194770 | ATP:guanidino kinase (Smc74), putative | | CAX73626.1 | | 90.08 | | - | | - | |

Table 3.2 (continued)

**Table S2: *S. mansoni* adult male worm excretory-secretory protein homologues.** *S. mansoni* WES proteins were subjected to similarity analysis using BLAST. Sequence alignments that showed a bit score higher than 30 and an E value lower than 1e-16 were considered homologs. ID – identification number at GeneDB or GenBank databases; (-) – no homologs found.
